# Immune Responses to Bacillus Calmette–Guérin Vaccination: Why Do They Fail to Protect against *Mycobacterium tuberculosis*?

**DOI:** 10.3389/fimmu.2017.00407

**Published:** 2017-04-05

**Authors:** Juan I. Moliva, Joanne Turner, Jordi B. Torrelles

**Affiliations:** ^1^Department of Microbial Infection and Immunity, College of Medicine, The Ohio State University, Columbus, OH, USA; ^2^Center for Microbial Interface Biology, The Ohio State University, Columbus, OH, USA

**Keywords:** tuberculosis, *Mycobacterium tuberculosis*, bacillus Calmette–Guérin vaccine, innate immunity, adaptive immunity

## Abstract

*Mycobacterium tuberculosis* (*M.tb*), the causative agent of tuberculosis (TB), is the current leading cause of death due to a single infectious organism. Although curable, the broad emergence of multi-, extensive-, extreme-, and total-drug resistant strains of *M.tb* has hindered eradication efforts of this pathogen. Furthermore, computational models predict a quarter of the world’s population is infected with *M.tb* in a latent state, effectively serving as the largest reservoir for any human pathogen with the ability to cause significant morbidity and mortality. The World Health Organization has prioritized new strategies for improved vaccination programs; however, the lack of understanding of mycobacterial immunity has made it difficult to develop new successful vaccines. Currently, *Mycobacterium bovis* bacillus Calmette–Guérin (BCG) is the only vaccine approved for use to prevent TB. BCG is highly efficacious at preventing meningeal and miliary TB, but is at best 60% effective against the development of pulmonary TB in adults and wanes as we age. In this review, we provide a detailed summary on the innate immune response of macrophages, dendritic cells, and neutrophils in response to BCG vaccination. Additionally, we discuss adaptive immune responses generated by BCG vaccination, emphasizing their specific contributions to mycobacterial immunity. The success of future vaccines against TB will directly depend on our understanding of mycobacterial immunity.

## Introduction

Tuberculosis (TB) disease, defined by symptoms cause by infection with *Mycobacterium tuberculosis* (*M.tb*), is the leading cause of death from an infectious agent (1, 2). *M.tb* continues to spread due to the existence of a large reservoir of latently infected individuals who can reactivate at any time (3). Clinical options available to combat TB include chemotherapeutic agents and the preventative vaccine *Mycobacterium bovis* bacillus Calmette–Guérin (BCG). BCG is a live-attenuated strain of *M. bovis* originally developed for its potential to prevent TB and not *M.tb* infection, an important distinction. BCG vaccination is highly effective at preventing TB-meningitis and extra-pulmonary disseminated TB; however, its efficacy against pulmonary TB (PTB) in different human populations (children, youth, adult, and elderly) varies. Some studies have shown 80% efficacy, whereas others have shown none (4, 5) [see Ref. (6) for a comprehensive list of BCG clinical trials]. Nonetheless, BCG is the most widely administered vaccine around the world for the prevention of *M.tb*-associated diseases (7).

The poor efficacy conveyed by BCG at preventing TB has been attributed to many factors, including human and mycobacterial genetics (8, 9), exposure to environmental mycobacteria (EM), coinfections with viruses and/or parasites, geographical location, and importantly, socioeconomic and nutritional factors (10–12). However, the fundamental question remains as to what constitutes sterilizing immunity to TB, and why BCG fails to confer this state. BCG is primarily believed to mediate immunity through the development of antigen (Ag)-specific memory T cells (13–15), which act quickly following a subsequent infection with *M.tb*. Indeed, this is the fundamental reason why BCG works against disseminated TB and TB meningitis (16, 17). However, why the same mechanism fails to prevent PTB remains poorly understood. Despite this, researchers across the world have continuously sought to modify and/or improve BCG. This began with genetic manipulations through deletions and insertions of genes from virulent mycobacteria that has today evolved into generating sophisticated multifunctional vaccine strains such as rBCG ΔureC:Hly^+^, the most promising vaccine candidate to replace BCG (5). Albeit conceptually and experimentally promising, the majority of these recombinant BCG vaccines fail at fully preventing the development of PTB (12).

It is still unclear why BCG fails to prevent primary infection, reactivation, and PTB, but it is widely accepted that generating long-term protective immunity is essential. Thus, developing a successful vaccine against *M.tb* infection or TB requires an understanding how immunity develops following BCG vaccination, and the roadblocks behind why protective immunity is not sustained. In this review, we explore the host innate and adaptive immune responses to BCG, and how these further influence the host response to *M.tb* infection and progression to TB. Finally, we discuss an important and commonly overlooked factor in BCG vaccine design, the influence of the human lung environment, and its consequences in directing the *M.tb* pathway of infection.

## Innate Immune Responses to BCG Vaccination

### Macrophages

Following BCG intradermal inoculation, resident epidermal macrophages interact with BCG *via* several pattern-recognition receptors (PRRs), including complement receptor 3 (CR3) (18) and toll-like receptors 2 and 4 (TLR2/4) (19). C-type lectin family receptors such as the mannose receptor (MR) and the macrophage inducible Ca^2+^-dependent lectin (MINCLE) receptor are expressed on macrophages (20–22), but direct interaction between them and BCG, and its subsequent outcome, has not yet been described. Because the peripheral lipid portion of the cell wall is very similar between BCG and *M.tb* (23), it is predicted that their ability to infect tissue macrophages will be similar. However, BCG’s first contact occurs with resident epidermal macrophages, whereas *M.tb* contact, in the majority of cases, occurs with resident alveolar macrophages (AMs). Differences in the mechanisms of Ag recognition, Ag uptake, Ag processing, and Ag presentation between these two types of resident tissue macrophages remains unclear and may contribute to the reasons behind why BCG is not fully protective. Thus, resolving this discrepancy will be important to identify if epidermal vaccination is sufficient to protect against lung disease. In fact, the effect of serum opsonization is often overlooked during BCG vaccination (24–26). This process is thought to be crucial in initiating immune responses to the BCG vaccine. As an example, the host opsonin factor H, a regulatory protein of the complement system that downregulates the alternative complement cascade, can bind to the BCG cell surface (27) and partially inhibit its uptake by epidermal macrophages. Macrophages infected by factor H opsonized BCG can secrete elevated amounts of pro-inflammatory cytokines, potentially driving an acute response (high IL-6/TNF) (27). Thus, factor H opsonization of BCG could be detrimental to the primary goal of BCG vaccination (to generate a strong T cell memory response). This is probably because opsonized BCG gets killed too quickly, reducing the amount of time Ag is available for presentation, and thus negatively impacting T cell proliferation and recruitment to the site of infection. Like factor H, there are other serum opsonins in the epidermal tissue and thus, further studies are necessary to assess their effects in the generation of immunity to BCG.

From the mycobacterial perspective, the TB field assumes that BCG cell wall components will interact in a similar fashion with macrophage receptors as *M.tb* cell wall components do (28). However, differences exist, such as in the case of the mannose-capped lipoarabinomannan (ManLAM) of *M.tb* vs. BCG. The degree and pattern of mannose capping and fatty acid content in *M.tb* ManLAM vs. BCG ManLAM differs (28) and thus, it may dictate how this molecule is differentially recognized by macrophage receptors such as the MR, TLR2 (dimerized with TLR1 or TLR6), or TLR4, generating different immune responses that subsequently may affect Ag presentation. In fact, BCG macrophage stimulation *via* TLR2 or TLR4 drives differences in pro-inflammatory responses, T cell proliferation, and IFNγ secretion *in vitro* and results in differences in bacterial burden in the lung *in vivo* (19). Indeed, prolonged Ag stimulation of TLR2 in macrophages downregulates the expression of major histocompatibility complex (MHC) class II and affects MHC I Ag cross processing, thus, reducing Ag presentation to T cells (29). It is still not clear to which degree suppression of MHC I and II *via* TLR2 will negatively impact the protective immune response generated by BCG vaccination and/or against *M.tb* infection. In this regard, studies have shown the potential of introducing an adjuvant during BCG vaccination targeting TLR-7/9 signaling, which restores and/or increases the expression of MHC II in macrophages, thus enhancing their ability to present Ag (30).

From the host perspective, differences in tissue-resident macrophages (epidermal vs. lung) expressing different PRR types and levels (31, 32), as well as human polymorphisms in these receptors, could influence immune responses to BCG and subsequently its effectiveness. Studies performed using BCG and *M.tb* indicate that the nature of their cell wall components engaging a specific macrophage PRR determines the host immune response generated. For example in macrophages, engagement of ManLAM or higher-order phosphatidyl-*myo*-inositol mannosides (PIMs) to the MR drives an anti-inflammatory response; however, engagement of trehalose 6,6′-dimycolate (TDM) to MINCLE or engagement of lower-order PIMs to CR3 and/or TLRs results in pro-inflammation (28). Thus, the balance between pro- and anti-inflammatory lipids present on the BCG and *M.tb* cell wall is a factor to consider in the protective ability of BCG. Reducing pro-inflammation at the site of vaccination may be beneficial as macrophages will be exposed to the Ag for a longer period of time, and thus provide additional time for the generation of T cell immunological memory. If we consider that epidermal macrophages and AMs in the lung have and/or use different PRRs for BCG recognition that leads toward site-distinct mechanisms of Ag presentation, then a safe delivery of BCG into the lungs could be a way to optimize BCG efficacy.

Differences in intracellular trafficking and Ag processing could also explain the failure of BCG to confer long-term immunity. In fact, BCG, as well as *M.tb*, can block phagosome maturation (33–35) by engaging specific receptors, i.e., the MR (36), and although MHC Ag presentation and activation of the adaptive immune system is unaffected, it may be less than optimal (37, 38). In this context, exogenous induction of phagosome–lysosome (P–L) fusion (39), autophagy (12), and/or increasing phagosome leakage of Ag across the phagosome membrane (40), could enhance the Ag-presentation process and thus, BCG efficacy.

An important step forward in the development of a new, effective TB vaccine, focused on optimizing Ag presentation, came from the development of the rBCG ΔureC∷hly vaccine (41). This vaccine, when administered percutaneously, has superior efficacy than BCG by further reducing the bacterial burden in the lung of *M.tb*-infected mice. Recent studies also indicate that vaccination with BCG ΔureC∷hly increases macrophage apoptotic vesicle formation, thereby inducing more robust CD4 and CD8 T cell responses (42), as well as increasing the gene expression of “Absent In Melanoma 2.” This increases the autophagic pathway and inflammasome activation, which in turn improves control of *M.tb* infection (43). Thus, although P–L fusion is an important mechanism, modulation of apoptosis and autophagy can also offer novel avenues for vaccines against TB. Mechanistic studies such as these, focusing on the underlying factors behind enhanced immunity offer valuable insight into the requirements for efficient mycobacterial clearance.

Extending on the above findings, *ex vivo M.tb*-infected lung macrophages from BCG-vaccinated guinea pigs are shown to secrete larger amounts of IFNγ, TNF, and IL-12p40, highlighting their appropriate anti-mycobacterial response (44). Other supporting *ex vivo* studies indicate that AMs from BCG-vaccinated guinea pigs challenged with *M.tb* express significantly less IL-10 and more IL-12p40, compared to unvaccinated controls (45). This same study reported an elevated expression of MHC II on peritoneal macrophages from BCG-vaccinated guinea pigs (45). Thus, based on our current understanding of the requirements for mycobacterial immunity; immunity induced by the BCG vaccine results in responses to *M.tb* at the early phase of infection that directly impact macrophage function.

**Figure 1:**
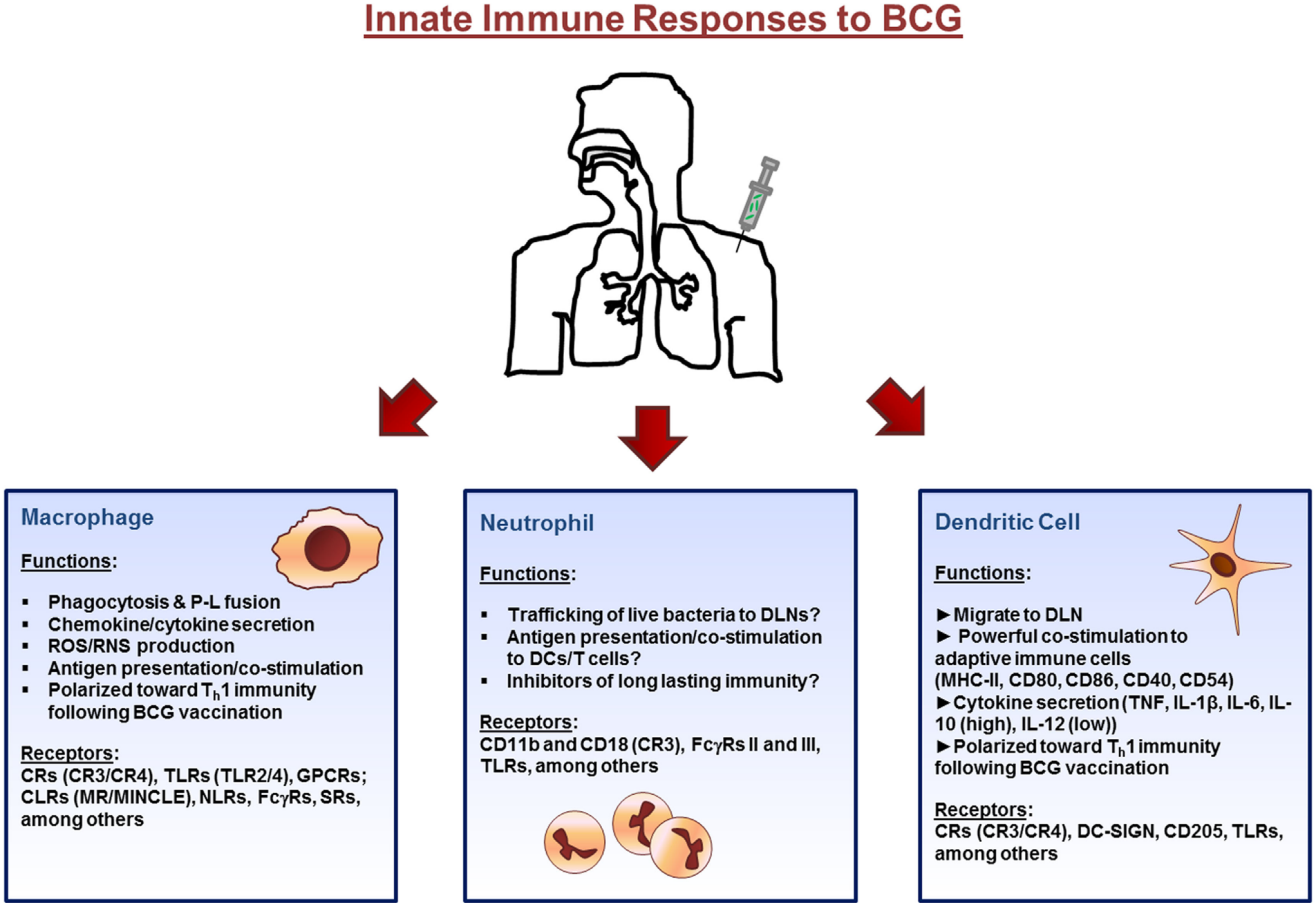
**Innate immune cell responses to bacillus Calmette–Guérin (BCG) vaccination**. The initial immune response to BCG occurs at the site of inoculation (usually the dermal layer of the skin) where resident macrophages and dendritic cells (DCs) interact with the bacillus *via* different receptors expressed on their surface. Macrophages and DCs phagocytose the bacteria initiating the innate immune response through the secretion of immunomodulatory components such as cytokines and chemokines. Bacteria are degraded *via* intracellular killing mechanisms and their peptides are trafficked to the plasma membrane along with major histocompatibility complex (MHC) class I and II where they are presented to cells of the adaptive immune system. Neutrophils also enter the site of inoculation and participate in the response. Finally, DCs, loaded with bacteria, and expressing antigen on their surface, home to draining lymph nodes. Abbreviations: P–L, phagosome–lysosome; ROS, reactive oxygen species; RNS, reactive nitrogen species; CRs, complement receptors; TLR, toll-like receptors; GPCRs, G-protein-coupled receptors; CLRs, C-type lectin receptors; MR, mannose receptor; MINCLE, macrophage inducible Ca^2+^-dependent lectin; NLRs, NOD-like receptors; FcγRs, Fcγ receptors; SRs, scavenger receptors; DLNs, draining lymph nodes; DC-SIGN, dendritic cell-specific intercellular adhesion molecule-3-grabbing non-integrin.

Altogether, there is a significantly large body of knowledge on the interactions between BCG and macrophages and how these responses can influence protective immune responses against *M.tb*. Receptor interaction between the macrophages and bacteria are critical in initiating the response, where interactions with different macrophage receptors can differentially modulate trafficking pathways and processing and presentation of Ag. However, additional studies determining the exact role of epidermal macrophages vs. AMs following BCG vaccination are needed before macrophage functions can be exploited to further improve vaccine development strategies. The innate immune response to BCG are highlighted in Figure 1.

### Dendritic Cells (DCs)

Dendritic cells are classified as one of the most potent APCs (46) and classically described as the modulators of cross talk between innate and adaptive immunity (47). Phenotypic and functional differences exist between DCs and macrophages; however, the primary difference is that DCs, in contrast to macrophages, migrate from tissues *via* the lymphatic system and enter the draining lymph nodes, where they present Ags to naïve T cells (48) leading to the induction of effector T cell responses (49, 50). As DCs migrate, they mature by upregulating MHC II and CD80, CD86, CD40, and CD54 costimulatory molecules, all involved in the activation of adaptive immune cells (48, 51). Several phagocytic receptors on DCs recognize BCG, including CR3 (CD11b/CD18), CR4 (CD11c/CD18), dendritic cell-specific ICAM-3 grabbing non-integrin (DC-SIGN, CD209), and DEC-205 (CD205) (52–54). Of all these receptors, DC-SIGN is possibly one of the most important as neutralization of DC-SIGN with antibodies (Abs) inhibits the interaction of BCG with DCs by 80% (51). Signaling receptors such as TLR2 and TLR4 are also shown to be involved in DC activation and maturation by their interaction with BCG cell wall components (i.e., mAGP complex) (51, 55, 56).

Following BCG vaccination epidermal DCs initiate adaptive immune responses *in vivo* by trafficking from the site of inoculation to draining lymph nodes where they present Ag to adaptive immune cells. In this context, migratory DCs display an EpCAM^low^CD11b^high^ phenotype and are capable of priming CD4^+^ T cells *via* interleukin-1 receptor and myeloid differentiation primary response gene 88 signaling pathways (57). However, most of our understanding of DCs and BCG immunology is from studies conducted *in vitro*. Stimulation of DCs with BCG *in vitro* induces homotypic aggregation, upregulation of surface Ag presenting molecules, downregulation of endocytic activity, and release of TNF (58), implying DCs initially respond to BCG. However, it is difficult to discern whether these responses are adequate for optimal immunity. BCG can be engineered to enhance DC activation by the addition of *M.tb* genes encoding specific mycobacterial proteins into BCG (59), suggesting that DC responsiveness to BCG may not be optimal. Though immunity can be improved by augmenting DC activation, we still lack a fundamental understanding of the exact interactions or processes that result in optimal immunological protection. From the perspective of the host, mice lacking specific genes (e.g., IFNγ, IL-12, and TNF) have been valuable in deciphering some of the requirements for effective immunity to BCG and *M.tb*; however, they offer little insight as to the concentration and spatial organization required for their optimal efficacy. Transgenic mouse models allowing manipulation of specific gene products or conditional knockout mice allowing for spatial regulation could be useful in elucidating the necessary elements for optimal BCG efficacy in terms of space and time.

Stimulation of human DCs with BCG can increase surface expression of MHC-II, CD40, CD44, CD54, CD80, and CD86, all markers involved in DC activation, maturation, migration, and Ag presentation to T cells (60). DCs infected with BCG also secrete TNF, IL-1β, IL-6, IL-4, and IL-10, but not IL-12 (61). Strikingly, IL-4 is secreted at large concentrations indicating that its presence could shift immunity from a T_h_1 polarized response toward a T_h_2 response, potentially affecting BCG efficacy (62). However, recent studies disproved this hypothesis by showing that BCG-infected DCs cocultured with T cells are capable of inducing T cell proliferation and IFNγ secretion *in vitro*, the defined T_h_1 cytokine critical for the control of *M.tb* (63). However, IFNγ is shown to correlate poorly with protection against mycobacteria infection and disease (64, 65). Thus, more refined markers such as abundance of memory lymphocytes in the lung and multifunctional T cell diversity have arisen as better indicators of functional immunity (discussed below) (17, 66). Furthermore, reports indicate that *M.tb* hinders the efficiency and effectiveness of Ag presentation by macrophages and DCs (67, 68), whereas BCG is much more efficient at stimulating CD4^+^ T lymphocytes. Thus, although BCG may effectively stimulate the process of Ag presentation, the capacity of BCG-infected DCs to present Ag may be at a saturation point, and any further stimulation of the adaptive immune system may not be achievable (69). In this context, attempts such as BCG-expressing Fms-like tyrosine kinase 3 ligand [Flt3L, a hematopoietic growth factor that stimulates DC proliferation, and whose deletion reduces T cell responses by 50% (70)] did not show further enhancement of BCG efficacy against *M.tb* challenge. This finding is independent of early expansion of DCs and increased stimulation of BCG-reactive IFNγ-secreting T cells (71). As BCG may be less efficient in inducing DC maturation than *M.tb*, understanding the differences between them could lead to greater approaches in improving the interaction between BCG and DCs and thus, host immunity (72).

Several groups have exploited DC maturation to improve BCG immunity, with studies centered in modulating TNF and IL-10 dominating the field. TNF neutralization inhibits DC maturation post-BCG inoculation suggesting an important role for TNF in immunity generated by BCG (60). As a result, an adequate concentration of TNF is required for optimal DC Ag-presenting efficacy. BCG-infected DCs also secrete large quantities of IL-10, but not IL-12 (61). IL-12 is, however, highly expressed in IL-10^−/−^ mice following inoculation with BCG indicating the existence of an important balance between IL-10 and IL-12, which could activate/accelerate maturation of DCs (35) and serve as a potential host-directed therapy. These findings may explain why DCs from IL-10^−/−^ mice are more efficient at activating T cells in the draining lymph nodes (DLNs) (73). Thus, IL-10 may not only downregulate the migration of infected DCs to the DLN but also regulate IL-12 production and subsequent DC capacity to mature, diminishing T cell activation and proliferation, repressing the adaptive immune response generated by BCG (73, 74), and possibly affecting the development of immunological memory. Hence, downregulation of IL-10 and upregulation of IL-12 could to be important for generating an optimal immune response to BCG.

Apart from being powerful mediators of the immune response to BCG by initiating innate responses, DCs also translate information to the adaptive branch of the immune system and initiate the first steps that subsequently give rise to immunological memory. With this in mind, it is logical to explore mechanisms that can enhance DC–BCG interactions with intentions of amplifying BCG efficacy. Although the DC–BCG interaction appears to be highly efficient, it remains unclear why they fail in the lung. One could speculate that too much focus has been placed on DC responses to BCG *in vitro* and thus, it has misconstrued our notion of DC responses.

### Neutrophils

Circulating human neutrophils comprise approximate 60% of the blood cells, have a short half-life of 6–10 h, and are one of the first cells to respond to foreign molecules (75). Neutrophils secrete large amounts of chemokines and cytokines, priming long-lived phagocytes (76). Interaction of neutrophils with BCG increases their expression of adhesion markers CD11b and CD18, FcγRs II and III, and stimulates their upregulation of cytokines (e.g., IL-1α, IL-1β, and TGFβ) and chemokines (e.g., IL-8, CCL2, and CCL3) (77). How changes in neutrophil phenotype upon contact with BCG can influence the generation of protective immunity against *M.tb* is still unclear.

Neutrophils are capable of shuttling live BCG *via* the lymphatic system into DLNs and into the vicinity of DCs and T cells (78). The cross talk between BCG-infected neutrophils and DCs in this location delivers maturation signals to immature DCs and also assists DCs in their presentation of BCG Ags to prime CD4^+^ and CD8^+^ T cells (78–80). The delay in apoptosis observed in BCG-infected neutrophils supports the concept that BCG may use them as a vehicle to disseminate from the site of inoculation to DLNs (81). However, whether the host benefits from the neutrophil–BCG interaction remains unanswered. Additionally, the longer BCG remains within neutrophils increases the probability the bacteria will be killed by neutrophil intracellular mechanisms, possibly enhancing pathways involved in immune activation *via* Ag presentation (82). Studies conducted in the C3HeB/FeJ mice, which develop necrotic and hypoxic tubercle granulomas, found that BCG vaccination in these mice was associated with long-lasting immunity and reduced bacterial burden. The association was attributed to a reduction in the numbers of neutrophils (83). Thus, neutrophils may behave as a double-edged sword; on one hand, they seem to facilitate Ag presentation by shuttling live bacilli to the vicinity of DCs, but on the other hand, they may be responsible for preventing the development of long-lasting immunity. The balance between BCG’s intracellular survival and/or digestion, processing, and presentation by the neutrophil may impact DC maturation, the subsequent adaptive immune response (limiting the clonal expansion of Ag-specific effector lymphocytes), and the long-term establishment of a state of protective immunity.

Conversely, neutrophils can also direct activation of T cells in lymph nodes, and thus participate in the generation of adaptive immune responses (80, 84). A recent study indicates that neutrophils expressing CCR7 migrate to the lymph nodes in response to CCR7 ligands, CCL19 and CCL21. This migration seems enhanced during *in vivo* injection of complete Freund’s adjuvant (containing inactivated *M.tb* cell wall) in wild-type mice, but not CCR7^−/−^ mice (85). To further illustrate a role for neutrophils, studies using mice inoculated with BCG *via* the intranasal route show neutrophils entering the lungs in two waves. The first wave arrives between 1 and 3 days post-inoculation and could kill *M.tb*. The second wave of neutrophils enters the lung 3 weeks post-inoculation together with IFNγ- and IL-17A-producing T cells. This second wave of neutrophils is not associated with the ability to kill *M.tb*, and their movement into the lungs is dependent on the expression of IL-17RA (86). Subsequent studies show that *M.tb*-infected neutrophils are a prominent population in the lungs early during infection (87), and that *M.tb*-infected neutrophils promote adaptive immune responses to *M.tb* infection. Recent studies using BCG also demonstrate that neutrophils regulate inflammation *via* the secretion of IL-10, impairing the control of *M.tb* growth during chronic infection (88); thus, further establishing the dual role for neutrophils during infection, being active in not only controlling *M.tb* infection but also regulating the inflammatory response generated during infection. Since neutrophils enter tissues much sooner relative to other host cells, they could be a critical mediator in clearance or persistence of mycobacteria. However, how the regulatory functions of neutrophils influence adaptive memory responses generated by BCG vaccination remains unanswered.

Altogether, neutrophils can have a wide range of effects in the context of BCG vaccination. On one hand, they may help facilitate adaptive immune responses by aiding in Ag presentation and shuttling live bacteria into the vicinity of professional APCs, but on the other hand, their presence and propensity to induce strong inflammatory responses may be detrimental to the tissue in which they reside, propagating the disease, and potentially inhibiting the development of long-lasting immunity. Indeed, it remains unclear whether neutrophil shuttling of BCG is beneficial or detrimental as they may ultimately be used as a vehicle from which to disseminate to other organs. Further studies as to the role of neutrophils and mechanism in which they may be involved post-BCG vaccination could shed some light on the complex behavior of neutrophils.

## Adaptive Immune Responses to BCG Vaccination

### T Lymphocytes

**Figure 2:**
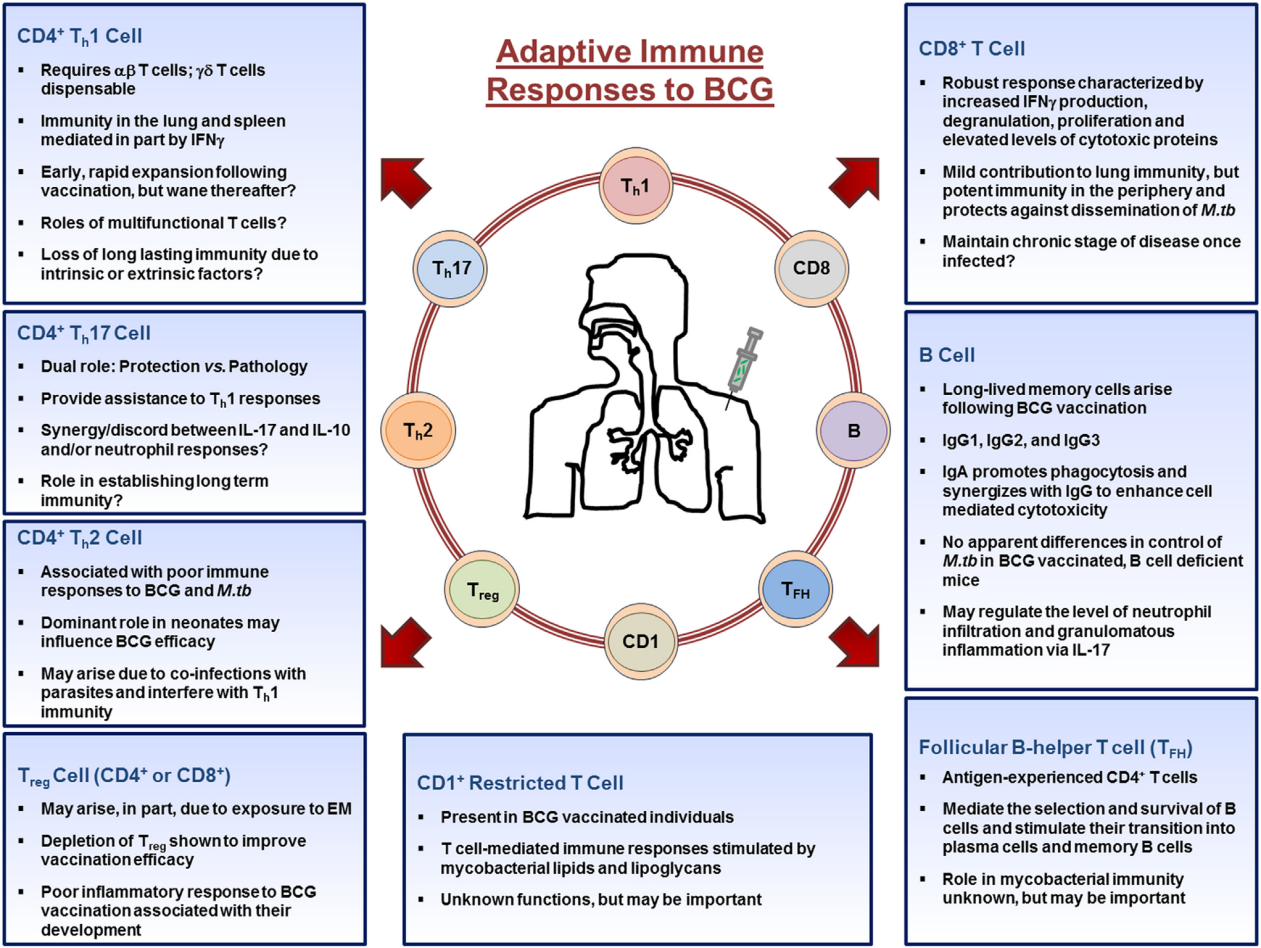
**Adaptive immune cell responses to bacillus Calmette–Guérin (BCG) vaccination**. Upon entering the lymph nodes, dendritic cells stimulate CD4^+^, CD8^+^, CD1^+^-restricted T cells, T_FH_, T regulatory cells, and B cells. CD4^+^ and CD8^+^ T cells migrate out of the lymph nodes toward the site of inoculation and provide the necessary stimulation to innate cells. CD4^+^ T cells differentiate into T_h_1, T_h_17, or T_h_2 cells depending on the stimuli present in their microenvironment and aid in the activation of macrophages, whereas CD8^+^ T cells mediate their functions by lysing infected cells or by secreting cytokines. B cells differentiate into antibody producing plasma cells or memory B cells. Throughout the process, memory cells arise from those that responded to the infection and populate peripheral organs, such as the lung. Together, the cells of the adaptive immune systems orchestrate the immune response in an attempt to establish mycobacteria immunity. Abbreviations: EM, environmental mycobacteria.

T cell responses arise in parallel through engagement of the T receptor with foreign Ag presented by Ag-presenting cells (89). With few exceptions, all vaccines stimulate the proliferation of CD4^+^ helper T cells (T_h_) and CD8^+^ cytotoxic T cells (T_c_) (14). In the context of BCG immunity, helper T cells primarily differentiate into two distinct classes of effector cells during an immune response to vaccination: Th1 cells identified by the production of IFNγ, and Th17 cells identified by the production of IL-17A, though IL-4 producing T_h_2 cells can also be generated (90). Other cell subsets such as T regulatory cells (T_reg_) and CD1-restricted T cells also arise following BCG vaccination, albeit to a lesser extent. BCG also induces cytotoxic T cells, whose main function is to lyse infected cells through osmotic disruption (91). It is clear that both CD4^+^, and to a lesser extent CD8^+^, T cells are critical for protection against *M.tb* infection; experimentally highlighted using mouse knockout models (92–95) supporting a dominant role for T cells as the main effector cells following immunization with BCG. The adaptive immune responses to BCG are highlighted in Figure 2.

#### CD4^+^ and CD8^+^ T Cells

Mouse models have confirmed that α/β T cell receptor expressed by CD4^+^ and CD8^+^ T cells and MHC I and II are necessary for control of mycobacterial infections (96, 97), highlighting a dominant T cell response to BCG. Adoptive transfer studies provide the best evidence that protective T cell mediate immunity is generated by BCG. The transfer of CD4^+^ or CD8^+^ T cells from BCG-vaccinated mice into *rag1^−/−^* mice, an *in vivo* model deficient for both B and T cells, show that CD4^+^ T cells are necessary to reduce bacterial burden in the lung and spleen, while CD8^+^ T cells control bacterial burden only in the spleen. These results implicate CD4^+^ T cells as the main effector cell generated by BCG in the lung, and also highlight the importance of CD8^+^ T cells in preventing dissemination (98–101), potentially making them the main effector cell responsible for preventing miliary TB and TB meningitis. However, despite the fact that CD4^+^ T cells reduce BCG burden in the lung, BCG is not eliminated, indicating that their effector functions in the lung may be limited. BCG vaccination followed by *M.tb* challenge confirms that in the absence of CD4^+^ T cells, CD8^+^-specific T cells are able to reduce *M.tb* bacterial burden in the lung at later time-points postinfection, supporting the importance of CD8^+^ T cells during the later phases of the disease (98, 102). These data demonstrate that BCG can stimulate protective CD4^+^ and CD8^+^ T cells and also suggest that CD4^+^ T cells are not as efficient as their CD8^+^ T cell counterparts when it comes to clearing a mycobacterial infection in a tissue-specific context, or perhaps the unique environment of the lung makes it difficult for CD4^+^ T cells to assert their functions. The answer could also lie in the pathogen itself as *M.tb* possesses many virulence factors that are not present in BCG that may be used to inhibit CD4^+^, but not CD8^+^, T cell responses.

The effector function of CD8^+^ T cells following BCG vaccination has been characterized to a lesser extent than CD4^+^ T cells (65). Whereas CD4^+^ T cells mediate their function by primarily activating cells of the innate immune system through engagement of costimulatory molecules on innate cells, CD8^+^ T cell responses, though similar, are not as capable of this function (98). Although there is a widely held view that BCG induces poor CD8^+^ T cell responses *in vivo*, human studies line up with *in vitro* studies showing that BCG vaccination induces robust Ag-specific CD8^+^ T cell responses characterized by increased IFNγ production, degranulation, proliferation, and elevated levels of cytotoxic proteins (103). However, the mechanism by which CD8^+^ T cells contribute to the efficacy of BCG, such as cytotoxic function, stimulation of other cells *via* cytokines, chemokines, or microbicidal molecules, remains elusive. One mechanism that has received little attention is the ability of CD8^+^ T cells to induce apoptosis of cells *via* the FasL–Fas pathways, whereby CD8^+^ T cells lyse target cells expressing Fas (104). By inducing apoptosis of infected cells, other healthy innate immune cells can phagocytose the produced apoptotic bodies and further stimulate cells of the adaptive immune system. Thus, another explanation for the superior ability of CD8^+^ T cell to control dissemination of *M.tb* could lie in their ability to induce the FasL–Fas pathway in the spleen or liver, whereas apoptosis of cells in the lung is highly restricted to prevent excessive tissue damage that can lead to pulmonary failure. In this context, one could envision an engineered BCG vaccine that induces apoptosis of infected cells allowing for a more efficacious vaccine against *M.tb* infection.

One argument frequently linked to the poor efficacy of BCG is the age at which BCG is administered, usually at the time of birth. As neonates are exposed to various Ags when born, it is believed that T cells are biased toward a T_h_2 response to prevent excessive inflammation. As a T_h_2 response can be detrimental to mycobacterial immunity, BCG vaccination of neonates may not fulfill its potential (105–109). However, studies in human newborns and infants reported a T_h_1 biased response following BCG immunization similar to immunized adults (110, 111). These studies observed a high number of IFNγ^+^CD4^+^ T cells (112, 113), and also a significant population of CD4^+^ T cells negative for IFNγ but instead positive for TNF and IL-2 (114) indicating that BCG generates diverse and adequate immune responses required to contain *M.tb* in infants. Thus, it seems that poor efficacy of BCG against *M.tb* may not be due to the failure of generating Th1 cellular immunity at the time of vaccination. On the other hand, it is reported that cytokine and chemokine production differs in children vaccinated with BCG in Malawi and the United Kingdom (UK) (115, 116). These studies pointed out that Malawian infants produce more cytokines associated with T_h_17 and T_h_2 immunity compared to infants from the UK. An explanation for this could be the environment surrounding the BCG-vaccinated infants [e.g., parasitic coinfections that dampen protective immune responses (117), or EM (118)]. Indeed, oral tolerance to EM is a detrimental factor following intradermal BCG vaccination, an effect that could be overcome by vaccinating *via* the pulmonary route due to lack of tolerance to EM in the lung (119). However, virulent lipids on the mycobacterial cell wall (including *M.tb* and *M. bovis* BCG), particularly TDM, have prevented the use of pulmonary vaccination. TDM is a highly toxic glycolipid that induces the formation of granulomas and a significant contributor of immunopathological damage to the lung during mycobacterial infections (120). Thus, another approach would be to focus on the bacteria itself and the biochemical properties associated with its cell wall that enable it to subvert host immunity. Despite whether BCG is efficacious or not at generating a strong T_h_1 response at birth, immunity appears to wane as we age. Thus, the problem of sustaining anti-mycobacterial immunity arises. Careful analysis of the mechanisms that protect against *M.tb* in the first decades of life post-BCG immunization could yield valuable clues to extend the duration of immunity generated by this vaccine.

#### CD4^+^ T_h_17 T Cells

T_h_17 responses in the lung are associated with both increased protection against *M.tb* infection (121) and exacerbated pathology (122). On one hand, repeated BCG vaccination exacerbates the influx of granulocytes/neutrophils into the lung in an IL-17A-dependent manner leading to extensive immunopathology. On the other hand, IL-17A is required to sustain IFNγ responses by CD4^+^ T cells in the lung. In mice, BCG stimulates T_h_17 responses within the lung (123). Thus, the presence of IL-17A in the lung may be important in generating an effective immune response that benefits the host (bacterial control with limited inflammation) or an immune response that ultimately damages the host (initial bacterial control with too much inflammation that subsequently leads to uncontrolled bacterial growth). Studies point out the role of IL-17A in the immune response to *M.tb* (124), particularly during initial granuloma formation (125). Interestingly, high concentrations of IL-17A that limit lung pathology are correlated to the presence of IL-10 (126). Although the relationship between IL-17A and IL-10 remains unclear, IL-10 appears having an immunosuppressive role during the generation of BCG immunity (127), and like IL-17A (121), IL-10 plays an important role during the initial stages of *M.tb* infection *in vivo* (128). The role of Th17 cellular responses in the context of BCG immunity remains uncertain, as indicated by recent studies demonstrating that the *M.tb* glycoprotein Rv1860/mpt32 (Apa or 45 kDa, present also in BCG) can downregulate T_h_17 and T_h_1 immune responses, abrogating BCG immunity against *M.tb* (129). However, this is in direct contrast to other studies where the same protein is shown to stimulate IFNγ-secreting CD4^+^ and CD8^+^ T cells (130) preventing the waning of BCG immunity, thus decreasing *M.tb* burden post-challenge (131). An explanation for these opposing results could lie in how the Rv1860 protein is utilized; in the former study, a recombinant BCG expressing Rv1860 was used to vaccinate mice, whereas in the latter, Rv1860 was administered as a booster in BCG-primed mice. This example highlights the complexity of the immune system and how certain mycobacterial proteins may be detrimental or beneficial in the development of the initial immune response depending on time and place and may or may not be required for optimal long-term immunity. In further support of Th17 cells as critical mediators of immunity to BCG, it has been shown that accelerated T_h_1 memory responses in the lung of BCG-vaccinated mice are dependent on IL-17A and IL-23 derived from Ag-specific memory Th17 cells, and that these lung-resident memory Th17 cells quickly respond to *M.tb* infection (121). With this in mind, BCG vaccination in unison with host-directed therapies that augment T_h_17 responses in the lung could be a powerful strategy to increase the efficacy of BCG.

#### T Regulatory Cells

T regulatory cells have also emerged as important players following immunization with BCG. BCG vaccination trials have partially associated BCG efficacy with the geographical location where the trial is conducted. Low BCG protective efficacy is reported in regions of the world closer to the equator, where EM are common. Thus, BCG protective efficacy could be affected by prior host T_reg_ cell development due to pre-exposure to EM (119). The importance of CD4^+^ T_reg_ cells mainly relies on studies showing that their depletion decreases *M.tb* burden in BCG-vaccinated mice (132). Similarly, BCG boosted with an Ag85 *M.tb*-protein construct (Ag85-Mpt64_190–198_-Mtb8.4) significantly decreases the number of T_reg_ cells and that their depletion correlates with reduced bacterial burden in the lung of *M.tb*-infected mice (133). Furthermore, human studies in BCG-vaccinated adults, who responded with strong immunization-induced local skin inflammation, showed significantly increased levels of protective multifunctional CD4^+^ T cells (134). However, BCG-vaccinated adults who developed mild local skin inflammation showed increased levels of regulatory-like CD8^+^ T cells (134). Thus, factors that influence vaccination-induced inflammation could affect the development of T_reg_ cells, for example, due to genetic polymorphisms within populations or their exposure to certain environments. If certain populations have a predisposition toward generating a strong T_reg_ cell response, BCG vaccination may be less effective. Thus, understanding genetic differences between human populations, and in particular changes brought forth by their living environment, could yield useful clues as to why BCG fails to protect them against *M.tb*. In fact, an important factor to consider in this matter is that many studies on EM are carried out by first exposing animals to EM followed by BCG vaccination. Since BCG is given at birth, exposure to EM likely occurs after BCG administration. Hence, the above studies highlight that a vaccine booster (BCG or other) after exposure to EM may not aid in the development of any further immunity where T_reg_ cells may play a role (135). Overall, T_reg_ cells may be detrimental to BCG vaccine efficacy against TB if there are pre-exposures to EM, thus blocking their function during BCG vaccination could be critical in inducing optimal immunity to *M.tb*.

#### CD1-Restricted T Cells

As the mycobacterial cell wall is primarily 80% lipid (136), studies have also focused on elucidating the role of CD1-restricted Ag presentation during BCG vaccination (137). BCG immunized humans harbor a pool of CD1-restricted CD8^+^ T cells recognizing BCG-infected DCs (138). Studies in guinea pigs support this finding, showing that BCG vaccination induces humoral and CD1-restricted cytotoxic T cell-mediated immune responses stimulated by mycobacterial lipids and lipoglycans (139). Perhaps these lipid-restricted CD8^+^, T cells mediate immune responses in peripheral organs such as the spleen and liver and are thus responsible for maintaining the chronic stage of the disease and/or help establish latent infection. This CD1-restricted CD8^+^ T cell population could also be the mediator that prevents disseminated TB and TB meningitis. Unfortunately, the absence of group one CD1 molecules (CD1a, CD1b, and CD1c) in mice (140) has made it difficult to elucidate the exact role of the CD1-restricted CD8^+^ T cell population to BCG immunity and during *M.tb* infection.

#### Mucosal-Associated Invariant T (MALT) Cells

Innate-like MR1 (non-classical MHC-1b)-restricted CD8^+^ T cells, called MAIT cells, are also defined as important players in mycobacterial immunity by potentially acting as early sentinels to *M.tb* infection (141). Despite individuals with TB having low levels of circulating MAIT cells (142), these cells respond to BCG stimulation by producing higher levels of TNF and IFNγ (143). Thus, further studies are necessary to clarify the positive or negative impact of MAIT cells in the context of BCG immunity.

#### Multifunctional T Cells

Evidence for other correlates of protective immunity rose with the discovery of multifunctional CD4^+^ T cells, which simultaneously produce multiple cytokines (usually TNF, IFNγ, IL-17A, and IL-2). The contribution of these cells to mycobacterial immunity have been thoroughly discussed elsewhere (144), but it is important to stress that multifunctional T cells do not always correlate with protective immunity (145), and their importance should be carefully evaluated. As an example, the MVA85A vaccine (modified vaccinia virus Ankara made to express Ag 85A from *M.tb*) is developed as an intranasal booster to BCG. BCG-vaccinated, MVA85A-boosted individuals have significant increases in multifunctional T cells compared to BCG alone leading many to speculate that MVA85A would enhance the efficacy of BCG. However, further studies revealed that with or without the MVA85A booster, BCG-vaccinated individuals are equally susceptible to TB (145). Thus, multifunctional T cells may be a part of the puzzle (146), but may not contribute so significantly to protective immunity against TB as initially thought.

Overall, these findings reveal the complex nature of vaccinating with a live multifaceted organism such as BCG, where it is difficult to target only one branch of the immune system and generate effective, long-lasting immunity that prevents *M.tb* infection and the development of TB. Although the necessity of CD4^+^ T cells is without question, perhaps the reason why immunity fails in the lung is not due to poor CD4^+^ T cell responses *per se*, but rather due to inhibitory mechanisms in the lung preventing immunopathology. As highlighted above, the major role for CD8^+^ T cells appears to be in maintaining the chronic phase of the disease and seems to be particularly important in peripheral organs. Thus, it may not necessarily be that BCG is an inneffective vaccine; it may be that the lung inhibits BCG from living up to its full potential. Defining the contributions, whether good or bad, of other adaptive immune cell subsets (T_h_2, T_h_17, T_reg_, CD1-restricted, multifunctional, etc.) is also important. Additionally, variability in human genetics and the geographical environment in which we reside may have consequences on our ability to mount immune responses to vaccines and should be considered when designing future trials to test new TB vaccines. The problem of understanding the adaptive immune response to *M.tb* is further complicated by animal models that do not exactly recapitulate TB in humans. *M.tb* has evolved to infect humans and thus, humans may be the only species possessing the key to elucidating the mechanisms responsible for clearance of mycobacteria.

### B Lymphocytes

The generation of long-lasting immunity to most pathogens by current effective vaccines relies on long-lived humoral immune responses that mediate protection *via* Abs (14). The BCG vaccine is a strong inducer of humoral immunity; however, due to the intracellular nature of *M.tb* infection, the potential of Abs as significant contributors to protective immunity has been largely disregarded. However, new evidence has emerged identifying a potential role for Abs and B cells in the immune response generated by BCG vaccination (147, 148). Researchers have begun to unravel a more significant role for B cells than solely Ab production in the context of mycobacterial infections (149, 150).

#### Ab Responses to BCG Vaccination

Although *M.tb* intracellular *modus vivendi* within host cells limits Ab function, rapid and effective Ab opsonization of *M.tb* prior to entry into phagocytes could be a mechanism resulting in clearance by innate immunity. Studies have demonstrated that BCG vaccination induces long-lived mycobacteria-specific memory B cells in healthy individuals (151), but details about their role in establishing immunity to BCG remain unclear. Early studies looking at Abs following BCG vaccination show agglutination in serum from BCG-vaccinated patients when incubated with *M.tb* Ags (152). Following this discovery, the humoral immune response following BCG vaccination (153–156) is associated with high levels of Ab production (157, 158), specifically linked to a progressive increase in the levels of immunoglobulin (Ig) M and IgG Ab isotypes IgG_1_, IgG_2_, and IgG_3_, the latter being induced by T_h_1 cytokines (159, 160). Overall, these studies identified robust humoral immune responses following BCG immunization, indicating that the BCG vaccine itself can reliably induce Ab responses. In the context of *M.tb* infection, low IgM and IgG levels are also partially linked to TB susceptibility (161, 162); an indication that perhaps a rapid humoral immune response in the lung may prevent *M.tb* infections by mitigating *M.tb* entry *via* portals that favor its establishment [i.e., *M.tb* ManLAM/host the MR (36)].

IgA is the most abundant Ab in the lung mucosa comprising approximately 30% of the total (163). Studies of IgA during BCG vaccination showed that IgA deficient (IgA^−/−^) mice have increased susceptibility to BCG infection and reduced production of both IFNγ and TNF in their lungs (164). IgA is shown to have a dual role, increasing phagocytosis of microbes and synergize with IgG to enhance cell-mediated cytotoxicity by effector T cells and also blocking IgG pathogen opsonization (163), potentially preventing interactions with Fcγ receptors. Thus, proper levels of IgA in the lung mucosa may determine the initial establishment of infection with *M.tb*. This finding is supported by other studies using polymeric IgR knockout (pIgR^−/−^) mice, where pIgR mediates active transport of dimeric IgA (165). As in the case of IgA^−/−^ during BCG infection, pIgR^−/−^ mice are also more susceptible to *M.tb* within the first 3 weeks of infection, mainly due to an increased influx of neutrophils to the site of the infection. Thus, the development of a new recombinant BCG strain capable of inducing the production of IgA-committed memory B cells may provide a good strategy to consider for the development of a mucosal vaccine. Given that we do not yet know how long *M.tb* remains in an acellular state following initial encounter with a host, Abs could mediate the very first interaction between the host cells and *M.tb*. As an example, opsonization of *M.tb* with Abs and subsequent entry *via* Fcγ receptors could result in increased bacterial killing compared to entry of *M.tb via* the MR (36). Given the large number of individuals exposed to *M.tb*, it is surprising that few develop the disease and instead remain healthy (PPD and/or QTF negative). BCG could be engineered to increase the presence of protective neutralizing/opsonizing Abs in the lung mucosa, so when *M.tb* is encountered it is quickly neutralized. Further research on Abs in the context of *M.tb* vaccine design could answer some of these pressing questions.

#### Cell-Mediated Responses to BCG Vaccination

The specific role of B cells has also been studied in the context of BCG vaccination (149) to a certain extent. In this regard, studies using BCG-vaccinated μMT mice [lacking one of the IgM μ-chain transmembrane regions and thus cannot produce mature B cells or secrete Abs of any isotype (166)] showed that these can retain the typical 1.0-log_10_ reduction in bacterial burden in their lungs following *M.tb* infection (167). This finding directly questions the role of B cells in the control of *M.tb* infection and suggests that B cells may not play a measurable protective role during BCG vaccination in mice. However, these studies do not completely rule out that BCG-induced Ab responses or B-cell-dependent costimulation could be targeted to prevent *M.tb* infection in the first place. In fact, a non-Ab-mediated role of B cells has recently emerged with the study of Follicular B helper T cells (T_HF_). T_FH_ are Ag-experienced CD4^+^ T cells found within secondary lymph node organs (e.g., spleen, lymph nodes) in the vicinity of B cell follicles (168). These cells are important in mediating the selection and survival of B cells and stimulate their transition into plasma cells and memory B cells (169). Recently, T_FH_ cells have emerged as important mediators in the development of BCG immunity. While studying the BCG ΔureC∷hly vaccine, it was discovered that the superior efficacy behind this vaccine correlates with higher levels of central memory T cells and T_FH_ cells (170, 171). Though promising, further research will reveal the importance of T_FH_ in the context of mycobacterial immunity.

There are still many unknowns regarding B cell immunity against *M.tb*, in particular in the context of BCG immunization. The role of Abs, though previously largely dismissed, could be targeted to prevent infection with *M.tb*. For example, by generating a long-lasting pool of *M.tb*-specific Abs within the lung mucosa, *M.tb* could be neutralized before it has the opportunity to encounter alveolar compartmental cells where it is shielded from opsonins. The cross talk between B and T cells could also play an important role in host defense against *M.tb*, including priming effective memory T cell responses. An important Ab-independent function for B cells is also plausible, as reported for other intracellular pathogens (172). Hence, whether B cells can be targeted by new vaccines to contribute *via* Ab-mediated processes or by interaction with other cells of the adaptive immune system to prevent *M.tb* infection requires further investigation.

## Adjuvants and BCG

Because we lack correlates of immunity to *M.tb*, research and development of new adjuvants that may enhance BCG immunity has been challenging [for details on TB vaccine adjuvants, see Ref. (173, 174)]. Whereas older adjuvant formulations relied on the use of cell wall extracts in liposomes or oil droplets (175, 176), modern adjuvants for TB vaccines target stimulation of PRRs including TLRs and MINCLE (174, 177). Adjuvants include cationic lipids, micro-/nanoparticles, toxin derivatives, CpG-containing DNA-based molecules, mycobacterial proteins conjugates, cytokines, and antimicrobial proteins (174). There are multiple studies examining adjuvant/adjunct molecules in combination with BCG to modulate innate responses and modify adaptive function boosting BCG efficacy (174, 178). In particular, lactoferrin, a host-secreted mediator that bridges innate and adaptive immune function in mammals (179), can act as an adjuvant with BCG protecting the lung alveolar integrity upon challenge with *M.tb* (180, 181). Adjuvants also make subunit vaccines more effective in boosting BCG (174), as a promising example is the IC31 adjuvanted H56 (182). This multistage vaccine is capable of boosting BCG efficacy delaying and reducing clinical disease in cynomolgus macaques challenged with *M.tb* and can prevent reactivation of latent infection (182). In general, adjuvants mediate their activities by (i) generating Ag depots, (ii) stabilizing Ag and protecting it from degradation, (iii) targeting Ag to specific cells, (iv) delaying or accelerating Ag uptake, (v) enhancing Ag presentation mechanisms, and (vi) directing stimulation of CD4^+^ and/or CD8^+^ T cells (174). However, the majority of studies designed to test adjuvant efficacy have targeted IFNγ responses, and as newer evidences suggest, IFNγ is an unsuitable marker of protection against *M.tb*. Although we continue to garner valuable knowledge on the mechanisms of action of adjuvants and how they can be improved, we must refine immunological correlates of protection if we are to use adjuvants to improve TB vaccine efficacy.

## The Quest for a Better Animal Model to Evaluate TB Vaccines

The lack of a validated animal model for TB vaccine development is a current critical issue in the field. There are several animal models routinely being used for TB vaccinology studies: mice, guinea pigs, and non-human primates (NHPs) are the most common ones.

Infection with *M.tb* in humans typically results in one of three outcomes. First, on rare occasions, *M.tb* can be quickly contained and the infection cleared with or without the assistance of the adaptive immune system. Although there is little evidence for this scenario, some individuals remain PPD negative despite exposure to *M.tb* (183). Second, in 90–95% of cases, the infection progresses into latency. During latent tuberculosis infection (LTBI), adaptive immune cells surround infected macrophages and form an enclosed structure called a granuloma. Individuals with LTBI can live their entire life without any symptoms of the disease. However, they have a 10% lifetime risk of developing active TB. Third, the remaining 5–10% of individuals progress directly to active TB and become contagious (184). In this context, the mouse model fails to replicate the natural progression of infection in humans. Although several groups are now using very low dose aerosol infections, the majority of studies still deliver 50–100 viable *M.tb* bacilli into the lung to reproduce natural infection in humans. In most mouse laboratory strains, *M.tb* replicates in the lung until Ag-specific T cells develop. At approximately 3 weeks post-infection, T cells enter the lung and stunt *M.tb* growth (185). *M.tb* bacterial burden peaks at approximately one million bacteria (6 log_10_) in standard mouse strains and remains at this level for an extended period of time before increasing at the end of life (186).

Outcome of BCG vaccination in mice and humans is also quite distinct. BCG-vaccinated mice are able to contain the *M.tb* infection sooner than naïve mice. Where it requires 3 weeks for mice to establish stable *M.tb* CFU, BCG vaccination shifts this pattern from 3 to 2 weeks. This allows the mouse to contain the infection more rapidly, establishing the bacterial burden at approximately 100,000 bacteria in the lung (5 log_10_) (187). This mycobacterial “immunity” has been attributed to immunological memory (13–15). However, immunity wanes across time. *M.tb* bacterial burden gradually increases back up to one million somewhere between 3 and 5 months post-infection. In humans, no studies have been conducted to evaluate the direct effects of BCG on the control of *M.tb* at early stages of infection mostly because it is extremely challenging to predict when infection will occur in a non-controlled setting. Thus, the current data on the efficacy of BCG come from clinical trials assessing whether individuals developed TB or not. Although it is not the focus of this review to discuss human clinical trial data on the effects of BCG vaccination, it is important to note that BCG efficacy against PTB in humans is predicted to be 60% and wanes with increasing age (12, 188).

The best relevant model for vaccine development that faithfully recapitulates human TB is the NHP. The two commonly used NHP models of TB research are the cynomolgus and rhesus macaques (189–191). Most research with BCG, however, has utilized the rhesus model. Rhesus macaques faithfully recapitulate human TB. They can develop asymptomatic TB, LTBI, progress to active TB, and even reactivate (192–195). The evidence clearly indicates that BCG can reduce *M.tb* bacterial burden in the lung, but does not eliminate it (196). Thus, data obtained using animal models suggest that BCG may in itself not prevent primary infection, but rather may exert its protective effects by containing *M.tb* and preventing progression to disease. Thus, the NHP is the ideal to model to screen TB vaccines. However, high costs, ethical issues, and challenges of animal handling have left the NHP as a last resort model for screening vaccine efficacy, and further, it is non-feasible as a high throughput model to test TB vaccine candidates.

## The Lung Environment and BCG Vaccination

Little is known about the role of the lung environment in determining the quality of immune responses generated during *M.tb* infection and the outcome of active or latent TB disease. The primary function of the lung is gas exchange and thus, the immune response generated within the lung is orchestrated to minimize inflammation. There is a delicate balance between generating a massive immune response that will be initially detrimental for the host and *M.tb* (i.e., cavities in active TB, destroying lung tissue and forcing the bacillus to accelerate replication, and abandon the dying host), or an immune response that will favor both (i.e., granuloma formation in latent *M.tb* infection, where both host and *M.tb* live in harmony). The lung environment has an impact on this balance. In this regard, the lung mucosa contains an array of homeostatic components (hydrolytic enzymes, complement proteins, surfactant proteins, antimicrobial enzymes, Igs, and many others) whose function is to maintain homeostasis of the lung (197, 198). All of these components are associated with the lung alveolar lining fluid (ALF) (199, 200). Studies from our laboratory have shown that some ALF components (i.e., hydrolytic enzymes or hydrolases) are capable of altering the cell wall of *M.tb* with two distinct outcomes, modifications on the *M.tb* cell wall exposing “*de novo*” motifs on the bacterium cell surface and the release of *M.tb* cell wall fragments to the lung milieu (201–204). The interaction of *M.tb* with human ALF reduces the amount of ManLAM and TDM by ~65 and ~40%, respectively, from the *M.tb* cell wall surface (201). It is likely that as *M.tb* is deposited in the alveolar space it will encounter ALF hydrolases that will modify its cell wall prior to encountering host cells. These *M.tb* cell wall alterations consequently alter *M.tb* recognition by human phagocytes (201–204) with subsequent impact on Ag processing and presentation. One question that remains is whether these human lung mucosa-induced alterations to the *M.tb* cell wall during its natural path of infection could explain the reason why the protective immune response generated by BCG vaccination is inadequate against PTB. For example, dominant cell wall motifs that drive Ag-specific B and T cell responses to intradermal administered BCG may be absent on ALF-exposed *M.tb* in the lung. Alternatively, BCG receptor-mediated uptake by epidermal-resident APCs may differ from those of *M.tb* in the lung due to newly exposed motifs on the *M.tb* cell surface by the action of human ALF.

To understand the role of the lung environment in the context of BCG vaccination, a valuable approach could be to determine how *M.tb* is modified in the lung prior to encountering alveolar compartment cells, how this affects its metabolism, and how it differs from the ones that BCG undergoes within the epidermis. Indeed, *M.tb* and BCG cell walls have few biochemical differences when grown on agar plates, but their cell wall and metabolism may differ as they are exposed to different microenvironments during infection (*M.tb*/lung) or vaccination (BCG/epidermis), with potential consequences in establishing effective or non-effective BCG immunity. Since the lung mucosa may play a significant role in how BCG may protect us against TB, the direct delivery of BCG into the lungs of humans may prove to be superior to the conventional systemic intradermal route of BCG administration. Whether it is by modulation of innate immune cell activity, activation of T cells, development of a rapid and robust Ab response, or by targeting specific components within the bacteria itself, BCG has potential to be further manipulated to enhance its efficacy against TB.

## Concluding Remarks

Bacillus Calmette–Guérin remains the only World Health Organization supported vaccine we have for the prevention of TB, yet lacks the ability to protect against the primary form of TB. We have highlighted the current knowledge in the field regarding innate and adaptive immune responses to BCG in the hope of stressing the importance of understanding immunological mechanisms that give rise to effective mycobacterial immunity. The importance of cross talk between the innate and adaptive branches of the immune system cannot be overstressed, yet the fundamental question of why BCG fails to fully protect against PTB remains. It could be due to coinfections (e.g., helminths, EM, etc.) preventing the full development of immune responses in the lung, or discrepancies between immune responses at the site of vaccine administration vs. the natural route of *M.tb* infection through the lungs. Perhaps it is not because BCG is poor at generating effective immune responses, but that the immunosuppressive status of the lung prevents it from doing so. Similarly, BCG could be at its saturation point, and thus further stimulation of the immune system would yield no added immunity. The answer could simply lie in shifting research efforts toward a more immunogenic route of vaccination. The literature suggests that intranasal and/or intratracheal vaccination with BCG is a more effective method to develop immunity against *M.tb*, yet no human studies have been published on this matter, mainly due to increases in pathology observed in the lungs using this delivery method. Thus, finding a way to decrease this inflammation in the lungs may open new avenues to explore direct mucosal vaccine delivery into the lungs. Efforts directed at exploring immunological events that occur following intranasal/intratracheal BCG vaccination and the status of immune cells within the lung could yield valuable answers.

We are now beginning to fully understand that *in vitro* studies do not always translate to *in vivo*. The development of more suitable animal models and implementation of −*omics* research could aid in the quest of finding a suitable replacement for BCG. Unfortunately, no effective vaccine yet exists for intracellular bacterial pathogens. A major question still remains: Is BCG poor at stimulating mycobacterial immunity or is *M.tb* simply adept at avoiding immunological responses against it? Furthermore, the majority of TB vaccine development research focuses on using laboratory strains and thus, does not assess vaccine efficacy against *M.tb* clinical isolates, with different degrees of virulence, heavily present in high TB burden regions. Studies directed at uncovering the mechanisms behind how BCG successfully primes, enhances, accelerates, and maximizes host immune recognition of *M.tb* should be prioritized. Furthermore, we must revaluate correlates of protection vs. correlates of risk and their implications in vaccine design. The TB field has placed much emphasis on the study of IFNγ responses and how they can be augmented, yet new evidence suggests that IFNγ is a better correlate of risk than of protection. Thus, we must begin to explore the contribution of other immune cells and factors and how they can be targeted to develop a more effective vaccine. We must also begin to design vaccine clinical trials that ask more refined questions about mycobacterial immunity and protection. Until we refine our understanding of immunity to mycobacteria, the development of a successful TB vaccine will remain a difficult task.

## Author Contributions

All authors equally contributed in the writing of this review.

## Conflict of Interest Statement

The authors declare that the research was conducted in the absence of any commercial or financial relationships that could be construed as a potential conflict of interest.
